# Effect of fibers on chloride transport in mortars under unsaturated and saturated conditions

**DOI:** 10.1039/d2ra08043e

**Published:** 2023-02-24

**Authors:** Danying Gao, Zhudi Cao, Lin Yang, Honglei Chang, Junru Li, Guowen Sun, Hongwei Liu

**Affiliations:** a Yellow River Laboratory, Zhengzhou University Zhengzhou 450001 China yanglin06142@zzu.edu.cn; b School of Water Conservancy and Civil Engineering, Zhengzhou University Zhengzhou 450001 China; c School of Qilu Transportation, Shandong University Jinan 250002 China hlchang@sdu.edu.cn; d School of Materials Science and Engineering, Shijiazhuang Tiedao University Shijiazhuang 050043 China; e Water Conservancy Bureau of Changyuan Changyuan 453400 China

## Abstract

The effect of steel fibers (0–1.5% by volume) and polypropylene fibers (0–0.5% by volume) on chloride transport in mortars under unsaturated and saturated conditions was investigated using a natural immersion method. Moreover, the micromorphology of the fiber–mortar interface and the pore structure of fiber reinforced mortars were detected using scanning electron microscopy (SEM) and mercury intrusion porosimetry (MIP), respectively. The results show that both of the steel fibers and polypropylene fibers have an insignificant effect on the chloride diffusion coefficient of mortars, no matter under unsaturated or saturated conditions. The incorporation of steel fibers has no obvious action on the pore structure of mortars, and the interfacial zone around the steel fibers is not a preferential path for chloride transport. However, the addition of 0.1–0.5% polypropylene fibers refines the pore size of mortars, and yet slightly increases the total porosity. The polypropylene fiber–mortar interface is insignificant, while the agglomerate of polypropylene fibers exists.

## Abstract 2

The effect of steel and polypropylene fibers on chloride transport in saturated and unsaturated mortars was investigated. Meanwhile, the pore structure of fiber reinforced mortar and the micromorphology of the fiber–mortar interface were analyzed.

## Graphical abstract

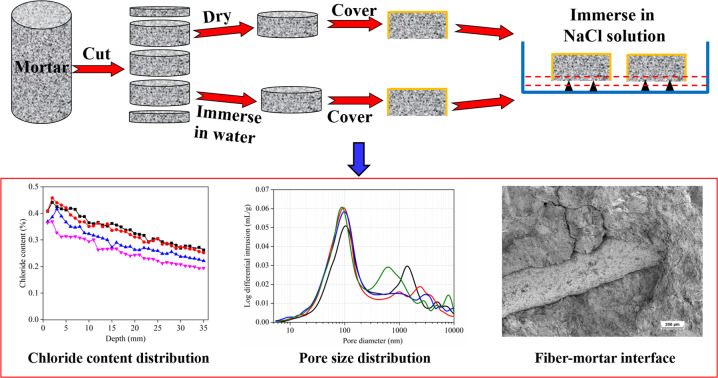

## 1. Introduction

Concrete is widely used in infrastructure construction owing to its excellent mechanical strength, durability, low cost, among other desirable properties.^1,2^ However, ordinary concrete has high brittleness and low fatigue resistance, and cracks easily under the action of internal and external factors, such as load, temperature and humidity changes, shrinkage, and so on. In severe environments, hazardous substances (*e.g.* Cl^−^) in the external environment readily enter the concrete through cracks, promoting internal steel corrosion, concrete expansion and spalling.^3–5^ In this context, adding fibers to ordinary concrete can effectively increase its toughness, tensile strength and flexural strength. Specifically, the randomly distributed fibers in concrete limit the expansion and cracking of concrete and improve its crack resistance.^6^

In recent years, various fibers, such as steel fibers, polypropylene fibers, and polyvinyl alcohol fibers, have been added to concrete to improve its performance.^7,8^ Among these, polypropylene fibers and steel fibers are the most widely used in concrete structures, because polypropylene fibers exhibit high crack resistance and steel fibers greatly improve the tensile strength of concrete. The addition of fibers inevitably affects the transmission of external hazardous substances while improving crack resistance of concrete and the mechanical properties. Some studies indicated that the addition of steel fiber improved the resistance to chloride permeability of concrete.^9^ In contrast, Hwang^10^ showed the chloride diffusion coefficient increased by 2–3 times upon adding steel fibers to ordinary concrete. The other researchers also obtained the similar conclusions.^11^ Nevertheless, Berrocal *et al.*^12^ indicated that the steel fibers did not greatly alter the chloride diffusion coefficient of uncracked concrete. Similarly, Frazao *et al.*^13^ indicated that the addition of steel fibers did not enhance the impermeability of concrete, but improve the flexural properties and energy absorption ability of cracked concrete. Ramezanianpour *et al.*^14^ investigated the impermeability of polypropylene fiber reinforced concrete by the electric flux method, and concluded that the polypropylene fibers improved the resistance to chloride permeability. However, Toutanji *et al.*^6^ found that the addition of polypropylene fibers reduced the resistance to chloride permeability, caused by the poor dispersion of fibers and poor adhesion to the matrix. Thus, the conclusions of previous studies on this topic are contradictory. Moreover, Behfarnia *et al.*^15^ compared the chloride resistance of polypropylene fibers and steel fibers, and reported that the effect of polypropylene fibers was better than the steel fibers at all dosages.

The inconsistent conclusions mentioned previously are mainly due to the use of diverse test methods. According to the measurement time, chloride migration can be analyzed by slow and rapid methods.^16^ Specifically, electrometric method that accelerates the migration rate of chloride ions in concrete by applying an external electric field, such as electric flux, rapid chloride migration (RCM), NEL Permit, AC impedance spectroscopy technology, *etc.*^17,18^ However, these methods are not suitable to test the chloride transport of steel fiber reinforced concrete, since the steel fibers are conductive and increase the electrical conductivity of concrete.^19^ Consequently, the experimental results cannot accurately reflect the migration characteristics of chloride ions in concrete. Thus, the use of rapid methods has been discredited by many scholars.^20–22^ Compared to the electrical acceleration method, the natural diffusion by immersing in salt solution is a reliable method to access the chloride diffusion of steel fiber reinforced concrete.^23–25^ Moreover, more than 90% of the concrete structures are in unsaturated state during the actual service, even though they are immersed in water for many years and difficult to be saturated.^26,27^ Thus, the saturation degree of concrete is an important factor influencing chloride transport, which should also be considered.^28^

This study focused on the effect of polypropylene fibers and steel fibers on chloride transport in mortars under unsaturated and saturated conditions. In addition, the effect of fibers on the fiber–mortar interface and pore structure of mortars were also investigated.

## 2. Experiment and method

### 2.1 Raw materials

In this work, PO 42.5 ordinary Portland cement (in Chinese standard) was used. The chemical composition of cement, as shown in Table 1, was measured by X-ray fluorescence method. The fineness modulus of natural river sand was 2.71. Hooked-ended steel fibers (SF) and polypropylene fibers (PPF) were used, and their physical parameters are shown in Table 2. NaCl powder (analytical reagent) and deionized water were used for the preparation of NaCl solution.

**Table 1:** Chemical composition of cement

Composition	CaO	SiO_2_	Al_2_O_3_	Fe_2_O_3_	SO_3_	MgO	Na_2_O	K_2_O	LOI^a^
Content (wt%)	62.54	19.29	4.97	3.40	3.99	3.85	0.25	0.85	0.86

a LOI: ignition loss for cement.

**Table 2:** Physical parameters of SF and PPF

Type	Appearance	Density (kg m^−3^)	Diameter (mm)	Length (mm)	Tensile strength (MPa)	Elastic modulus (GPa)
SF	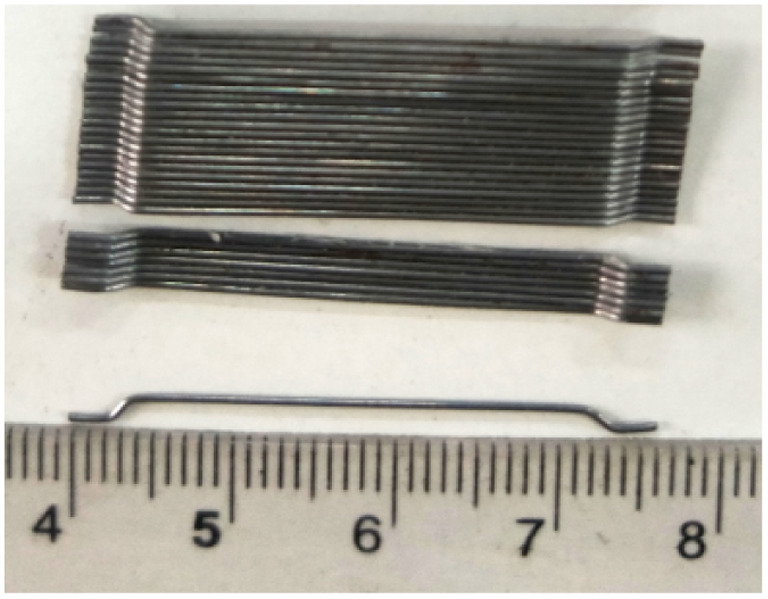	7800	0.55	35	1345	200
PPF	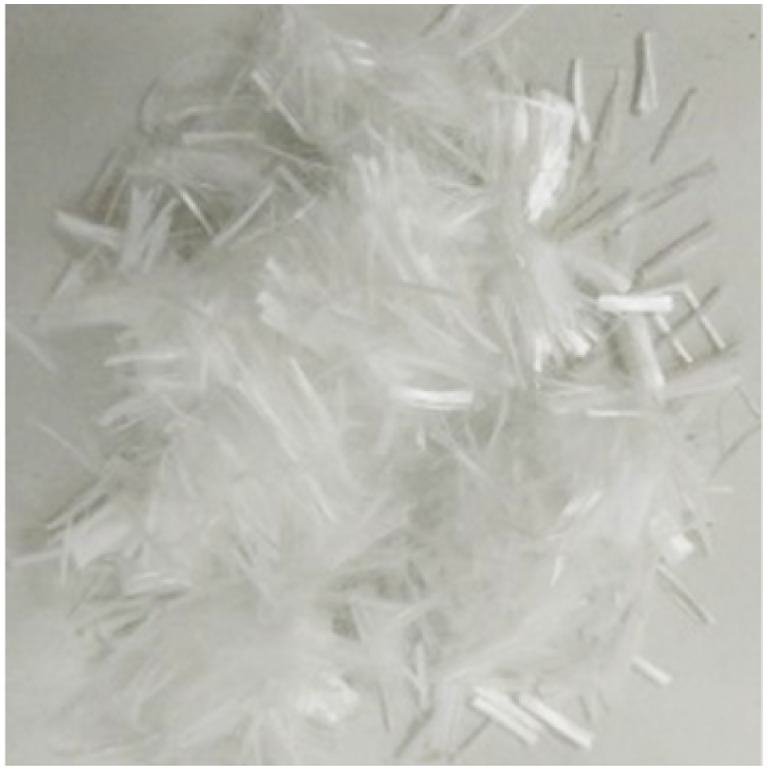	910	0.03	12	458	35

### 2.2 Mix proportion and specimen preparation

The water-to-cement ratio (W/C) was maintained at 0.5, and a constant mass ratio of cement to sand of 1 : 3 was used. For SF mortars, the amounts of steel fibers were 0, 0.5%, 1.0% and 1.5% (by volume fraction), respectively. Meanwhile, for the mortars blended with PPF, the volume fractions of polypropylene fibers were 0, 0.1%, 0.3% and 0.5%, respectively. For the investigation of chloride transport in mortars, specimens with a height of 200 mm and diameter of 100 mm were prepared and cured in water for 60 days.^29^ In addition, cubic specimens (100 mm in the side length) were molded and cured at 20 °C/95% RH for 28 days, and Table 3 shows their compressive strengths. It can be seen that the compressive strength of mortar increases gradually with the SF content increasing from 0% to 1.5%, while the addition of 0–0.5% PPF has no significant effect on the compressive strength of mortar.

**Table 3:** Mix proportion and compressive strength of mortars

Type of fiber	W/C	Cement to sand ratio	Fiber content (%)	Compressive strength (MPa)
SF	0.5	1 : 3	0	41.9
			0.5	42.6
			1.0	46.4
			1.5	48.2
PPF	0.5	1 : 3	0	41.9
			0.1	42.1
			0.3	43.6
			0.5	39.2

### 2.3 Experiment methods

#### 2.3.1 Chloride transport in mortars

**Fig. 1:**
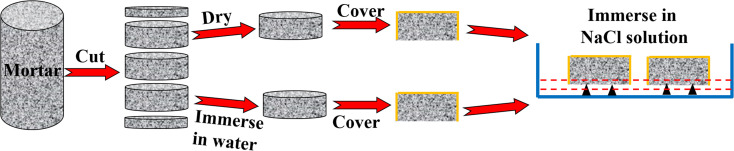
Sample treatment and chloride transport.

After curing, the samples were cut longitudinally into cylindrical samples with a thickness of 50 mm. Subsequently, the samples were divided into two groups: one group was dried in an oven at 50 °C until the weight did not change in twelve hours, while the other was immersed in water for 20 days for saturation. In order to ensure that the chloride ions transported in one-dimensional direction, the sides and one cross section of each cylindrical specimen were sealed with epoxy and the other was exposed to 3% NaCl solution, as shown in Fig. 1.

#### 2.3.2 Measurement of chloride content

After exposure to NaCl solution for 4, 8, and 12 months, the samples were fetched and the surfaces were cleaned. After that, the samples were ground layer by layer from the surface to the interior, and the thickness of each layer was 1 mm. The collected powder was sieved out of the large particles of >0.15 mm and dried in an oven at 105 °C for 48 h. For the mortars prepared with the addition of SF, the iron filings in the powder were removed using the magnet. The water-soluble chloride content in the powder sample was determined by the method of precipitation potentiometric titration, as follows: (1) about 1 g of powder sample was dissolved in 20 ml of deionized water, and the mixture was heated and boiled for 2 minutes; (2) after cooling to room temperature, the deionized water was added into the mixture to 50 ml, and then an automatic potentiometric titrator (ZDJ-4A, REX) was used to measure the water-soluble chloride content in the mixture; (3) the chloride content in the powder sample was calculated according to the following formula:1
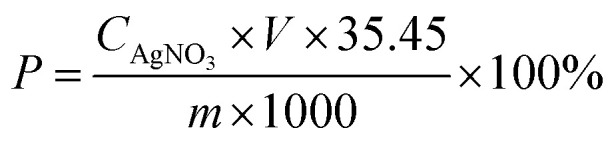
where *C*_AgNO_3__ is the concentration of the AgNO_3_ solution (mol l^−1^); *P* is the chloride content in the powder (%); *V* is the consumption amount of the AgNO_3_ solution (ml); *m* is the weight of the powder (g).

According to the chloride content distribution, Fick's second law with error function solution was used to calculate the chloride diffusion coefficient,^30^ as follows:2
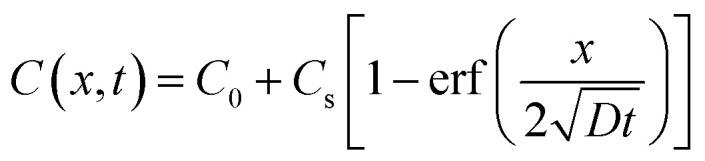
where *C*(*x*,*t*) is the chloride content at depth *x* and exposure time *t* (by mass of sample, %); *x* is the depth from the sample surface (m); *C*_0_ is the initial chloride content in the sample (*C*_0_ = 0 in this work); *t* is the exposure time (s); *D* is the apparent diffusion coefficient of chloride (m^2^ s^−1^) and *C*_s_ is the chloride content at the sample surface (%).

#### 2.3.3 Pore structure and micromorphology

Cubic samples with a side length of ≤8 mm were cut from the cured specimens and dried in the oven at 50 °C for one week. The pore structure was measured using mercury intrusion porosimetry (AutoPore 9500, Micromeritics, USA). To observe the fiber–mortar interface, the block samples were firstly cut from the mortar specimens, and also dried at 50 °C for one week. After that, the sample surface was sprayed with gold to increase the conductivity, then the micromorphology of the fiber–mortar interface was obtained using scanning electron microscope (Sigma500, ZEISS, Germany).

## 3. Results and analysis

### 3.1 Effect of steel fiber content on chloride transport

#### 3.1.1 Under unsaturated condition

**Fig. 2:**
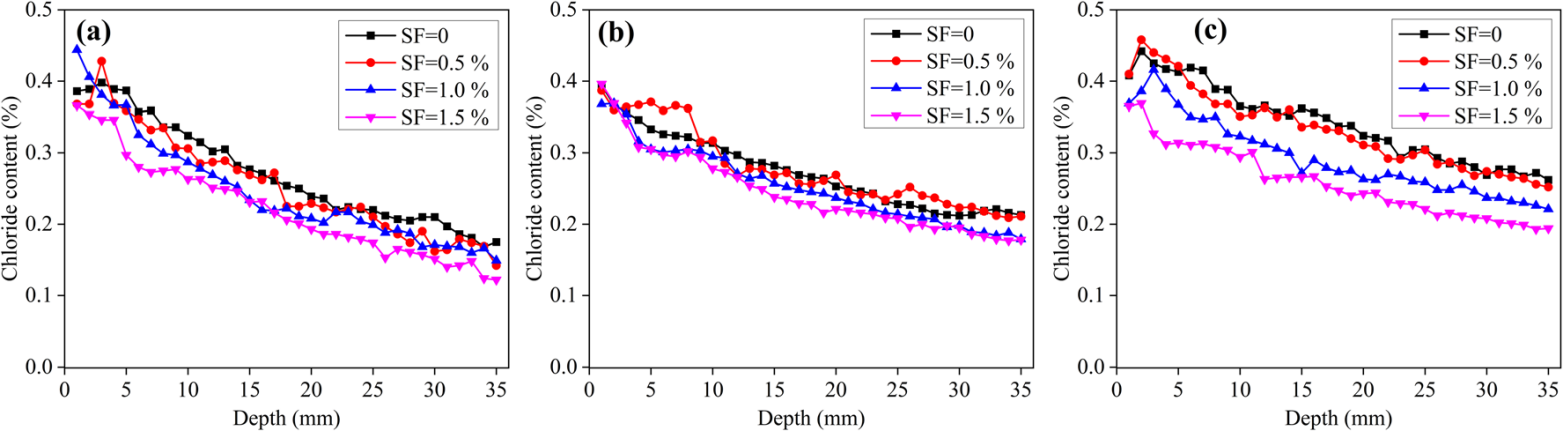
Chloride content distribution of unsaturated mortars with different SF contents immersed in NaCl solution for different time. (a) 4 months, (b) 8 months and (c) 12 months.

Fig. 2 shows the chloride content distributions of the unsaturated SF mortars immersed in NaCl solution for 4, 8 and 12 months. Under this unsaturated condition, chloride migration includes convection induced by the capillary suction and diffusion by the concentration gradient; besides, the former is the main way.^31^ As shown in Fig. 2, the chloride content has no significant difference at 4 and 8 months when the SF volume fraction increases from 0 to 1.5%; however, at 12 months, the chloride content has a slight decrease with the SF content increasing. According to the chloride content distribution, Table 4 records the chloride diffusion coefficients. The chloride diffusion coefficient of mortar has a slight decrease when the SF content increases from 0 to 1.5%; however, the variation is very small and can be ignored. Therefore, from these experiment results, the addition of steel fibers has insignificant influence on the chloride diffusion coefficient of the unsaturated mortar. In addition, the chloride diffusion coefficient has a slight decrease when the exposure time increases from 4 to 12 months, resulting from the continuous hydration of cement.^31^

**Table 4:** Chloride diffusion coefficient of unsaturated SF mortar

Exposure time (months)	SF content (%)	*C*_S_ (%)	*D* (×10^−11^ m^2^ s^−1^)	*R* ^2^
4	0	0.44	8.29	0.973
	0.5	0.39	7.27	0.955
	1.0	0.39	6.49	0.913
	1.5	0.35	6.36	0.960
8	0	0.36	7.36	0.956
	0.5	0.37	7.02	0.882
	1.0	0.35	6.04	0.958
	1.5	0.34	5.39	0.911
12	0	0.44	6.53	0.967
	0.5	0.43	5.88	0.943
	1.0	0.39	5.41	0.921
	1.5	0.35	4.84	0.951

#### 3.1.2 Under saturated condition

**Fig. 3:**
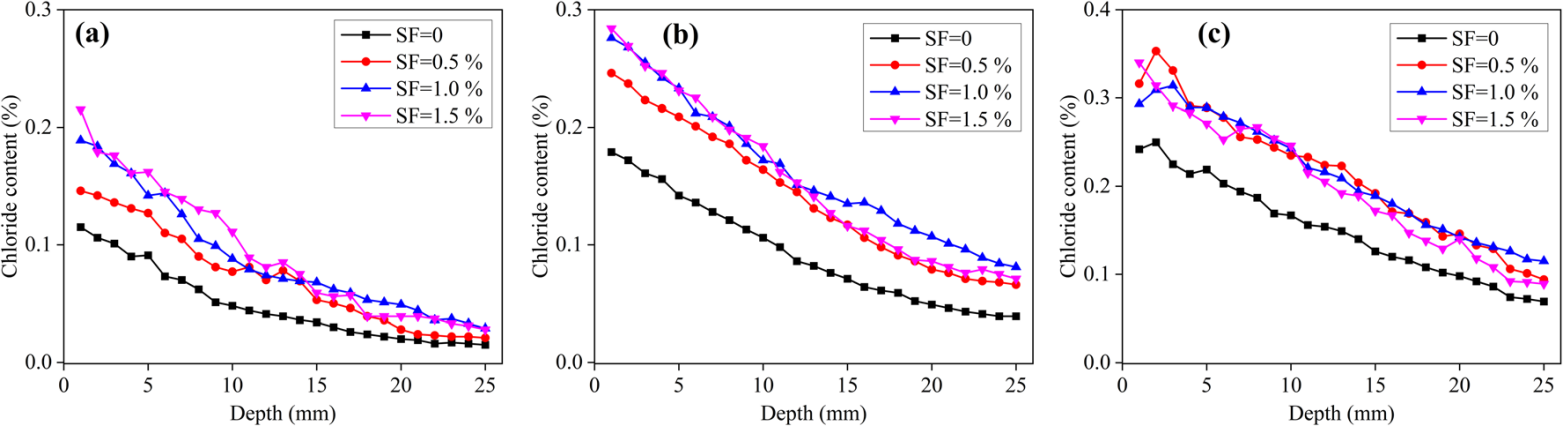
Chloride content distribution of saturated mortars with different SF contents immersed in NaCl solution for different time. (a) 4 months, (b) 8 months and (c) 12 months.

Fig. 3 shows the chloride content distributions of the saturated SF mortars immersed in NaCl solution for 4, 8 and 12 months. Under the saturated condition, chloride transport is controlled by the diffusion, caused by the concentration gradient.^31^ The chloride content of the mortars with the addition of SF has an increase, compared with the mortar without fibers. In addition, after 8 and 12 months of immersion, the chloride distributions of mortars with 0.5–1.5% SF have no obvious difference. Table 5 shows the chloride diffusion coefficients of the saturated mortars. From these results, the chloride diffusion coefficient has insignificant variation when the SF content increases from 0% to 1.5%, even though it has small fluctuations. That is, the addition of 0–1.5% SF has insignificant effect on the chloride diffusion coefficient of saturated mortars, which is consistent with the unsaturated mortars. Abrycki and Zajdzinski^32^ also concluded that the addition of varying dosages of SF did not significantly alter the penetration of chloride ions, using the method of non-steady state migration.

**Table 5:** Chloride diffusion coefficient of saturated SF mortar

Exposure time (months)	SF content (%)	*C*_S_ (%)	*D* (×10^−12^ m^2^ s^−1^)	*R* ^2^
4	0	0.12	9.15	0.979
	0.5	0.16	12.10	0.984
	1.0	0.19	13.0	0.961
	1.5	0.21	10.8	0.984
8	0	0.19	7.64	0.995
	0.5	0.26	9.89	0.995
	1.0	0.28	11.90	0.988
	1.5	0.29	8.88	0.990
12	0	0.26	8.10	0.994
	0.5	0.35	9.08	0.978
	1.0	0.33	10.80	0.984
	1.5	0.34	8.07	0.980

### 3.2 Effect of polypropylene fiber content on chloride transport

#### 3.2.1 Under unsaturated condition

**Fig. 4:**
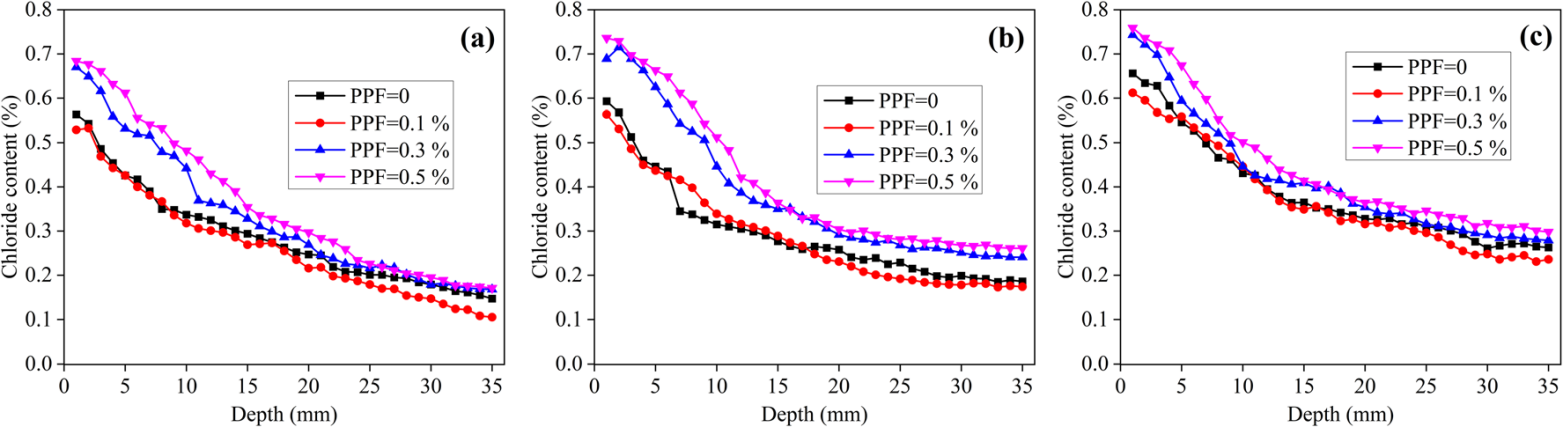
Chloride content distribution of unsaturated mortars with different PPF contents immersed in NaCl solution for different time. (a) 4 months, (b) 8 months and (c) 12 months.

Fig. 4 shows the chloride content distributions of the unsaturated mortars with different volume fractions of PPF, immersed in NaCl solution for 4, 8 and 12 months. When the PPF content increases from 0% to 0.1%, the chloride content does not change significantly; however, when the PPF content increases from 0.1% to 0.5%, the chloride content increases significantly. Table 6 shows the chloride diffusion coefficients of the unsaturated PPF mortars. When the PPF content increases from 0% to 0.5%, the chloride diffusion coefficient shows no obvious variation, regardless of the exposure time. In other words, under the unsaturated condition, the addition of 0–0.5% PPF has no significant effect on the chloride diffusion coefficient of mortar.

**Table 6:** Chloride diffusion coefficient of unsaturated PPF mortar

Exposure time (months)	PPF content (%)	*C*_S_ (%)	*D* (×10^−11^ m^2^ s^−1^)	*R* ^2^
4	0	0.51	4.36	0.944
	0.1	0.56	3.12	0.99
	0.3	0.65	3.33	0.958
	0.5	0.69	3.29	0.982
8	0	0.49	2.50	0.845
	0.1	0.51	2.09	0.934
	0.3	0.67	2.12	0.891
	0.5	0.71	2.13	0.88
12	0	0.59	2.31	0.891
	0.1	0.59	2.03	0.944
	0.3	0.66	2.05	0.886
	0.5	0.70	2.05	0.89

#### 3.2.2 Under saturated condition

**Fig. 5:**
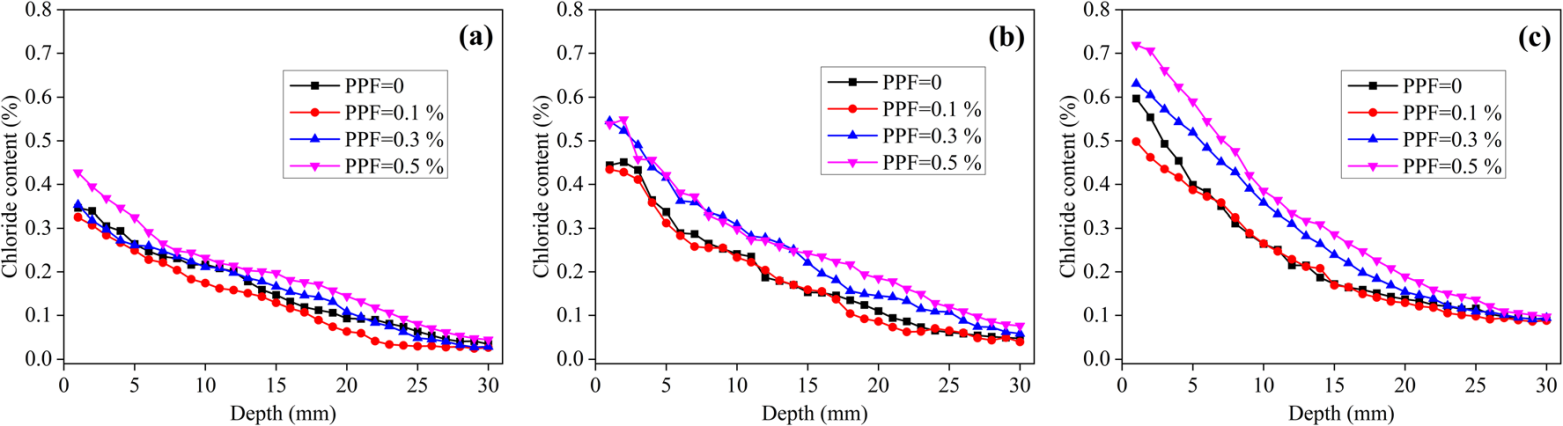
Chloride content distribution of saturated mortars with different PPF contents immersed in NaCl solution for different time. (a) 4 months, (b) 8 months and (c) 12 months.

Fig. 5 shows the chloride content distributions of the saturated mortars with different volume fractions of PPF, immersed in NaCl solution for 4, 8 and 12 months. Notably, the PPF content has no obvious effect on the chloride content distribution after 4 months of exposure. However, at 8 months, the chloride content of mortars with 0.3% and 0.5% PPF is higher than that of the mortars without and with 0.1% PPF. When the exposure time comes up to 12 months, the chloride content distribution exhibits a clear difference for the mortars with 0.1–0.5% PPF, even though the chloride content of mortars without PPF and with 0.1% PPF is similar. Consequently, the variation of fiber volume fraction has an effect on the distribution of chloride content in mortar. Table 7 indicates the chloride diffusion coefficients of saturated PPF mortars. Obviously, under the saturated condition, the addition of 0–0.5% PPF does not significantly affect the chloride diffusion coefficients of mortars after 4, 8 and 12 months of exposure, which is similar to the unsaturated mortar.

**Table 7:** Chloride diffusion coefficient of saturated PPF mortar

Exposure time (months)	PPF content (%)	*C*_S_ (%)	*D* (×10^−12^ m^2^ s^−1^)	*R* ^2^
4	0	0.35	16.40	0.992
	0.1	0.34	12.00	0.992
	0.3	0.35	17.60	0.98
	0.5	0.4	18.90	0.976
8	0	0.46	6.32	0.982
	0.1	0.45	6.06	0.983
	0.3	0.54	7.84	0.989
	0.5	0.53	9.86	0.975
12	0	0.55	4.47	0.942
	0.1	0.5	4.12	0.978
	0.3	0.66	4.82	0.993
	0.5	0.74	4.96	0.99

### 3.3 Microstructure of fiber reinforced mortars

#### 3.3.1 Pore structure

**Fig. 6:**
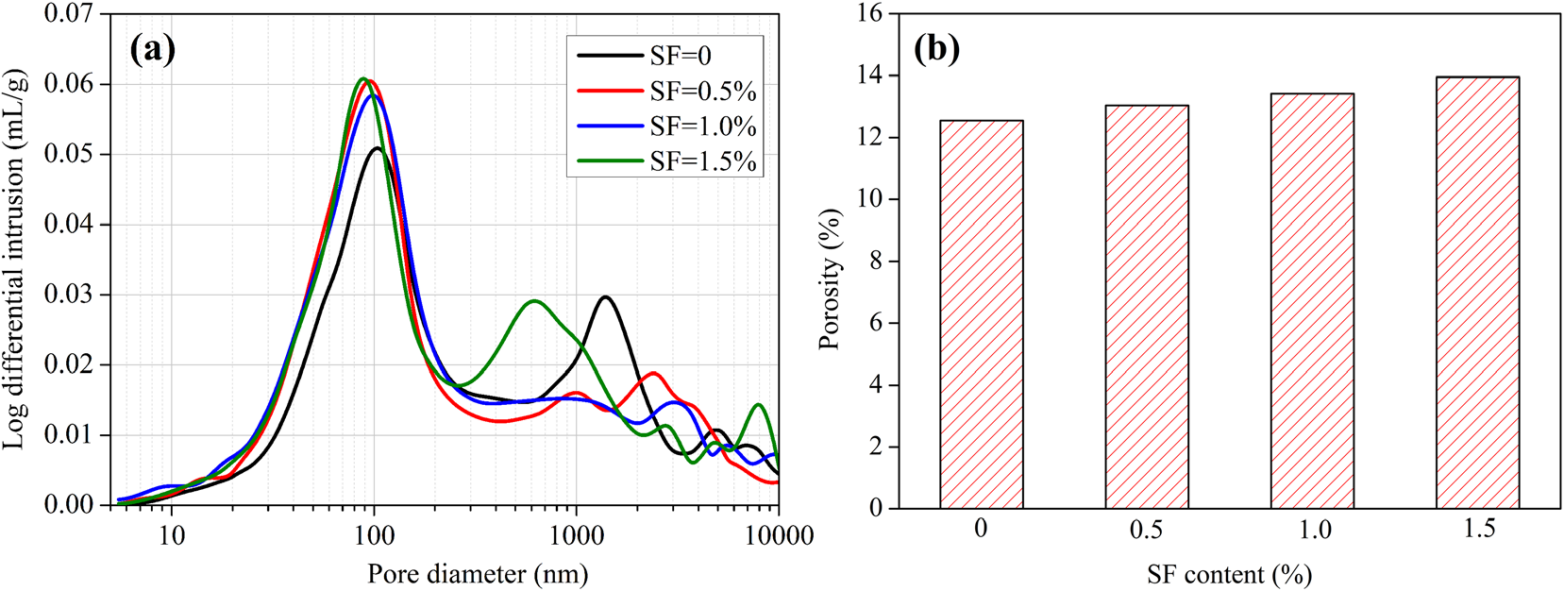
Pore structure of mortars with different contents of SF. (a) Pore size distribution, (b) total porosity.

The effect of fibers on chloride transport in mortars is complex, contributing to the change of pore structure and the fiber–mortar interface. Therefore, the pore structure of mortars with different volume fractions of fibers was measured to analyze the effect of fibers on chloride transport in mortars. Fig. 6 shows the pore structure of SF mortars. As shown in Fig. 6a, the pore size distribution of mortar has no significant variation as the SF content increases from 0% to 1.5%. In which, the average pore diameters of mortars are separately 129, 101, 97 and 107 nm when the SF contents are 0, 0.5%, 1.0% and 1.5%, respectively. This result illustrates that, compared to the mortar without SF, the addition of 0.5–1.5% SF decreases the pore size of mortars, but not significantly. As shown in Fig. 6b, with the SF content increasing from 0% to 1.5%, the total porosity of mortar increases from 12.55% to 13.95%. It also indicates that the addition of 0–1.5% SF has no significant effect on the total porosities of mortars. The steel fibers are evenly dispersed in the mortars, and they do not show obvious effect on pore structure of the mortars.^33^

As we know, the cement-based material is a typical porous material, since the pore size spans over three scales (micro, meso and macro) and several orders of magnitude (10^−9^–10^−3^ m), including gel pores, capillary pores and voids.^34^ Chloride transport in the cement-based material is governed by the pore structure (pore size, porosity, and connectivity) and interfacial transition zone, and usually the micro-pores provide continuous paths for chloride migration, no matter under saturated and unsaturated conditions.^35,36^ The addition of 0–1.5% SF has no significant effect on the pore structure of mortars, therefore, it does not obviously change the chloride diffusion coefficients.

**Fig. 7:**
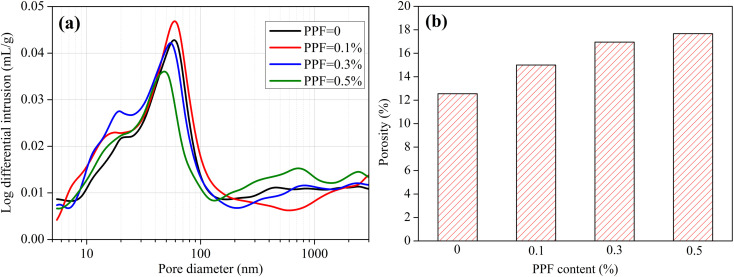
Pore structure of mortars with different contents of PPF. (a) Pore size distribution, (b) total porosity.

Fig. 7 shows the pore structure of PPF mortars. The PPF content has a significant effect on the pore structure of mortar. With the PPF content increasing from 0% to 0.5%, the pore size distribution has a small movement to the left (the smaller pore diameter), but not significantly. Especially, when the PPF content comes up to 0.5%, the proportion of small pores (<100 nm) has an obvious decrease while the proportion of large pores (200–1000 nm) increases slightly. As shown in Fig. 7b, the total porosity of mortar increases from 12.55% to 17.67% when the PPF content increases from 0% to 0.5%. Overall, adding PPF increases the total porosity of mortar but has a decrease in pore size. This is the reason that the addition of PPF has no significant effect on chloride transport in mortars under unsaturated and saturated conditions.

#### 3.3.2 Micromorphology of fiber–mortar interface

**Fig. 8:**
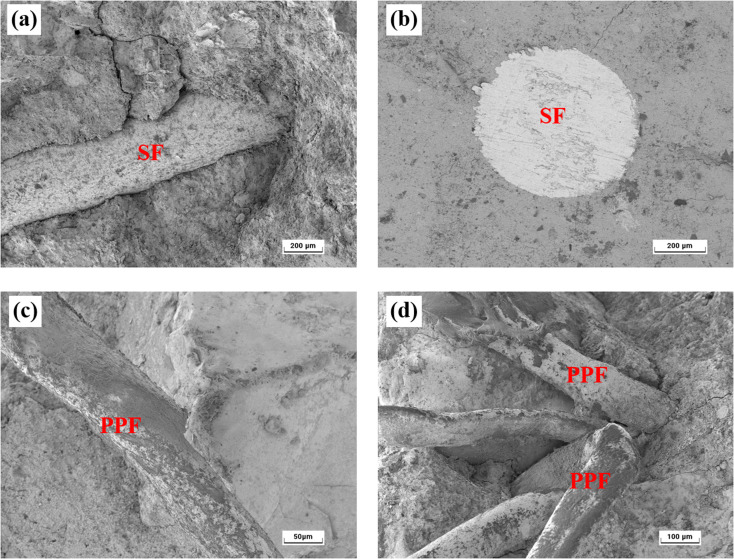
Micromorphology of fiber–mortar interface. (a) SF-mortar interface (SEM), (b) SF-mortar interface (BSE), (c) PPF-mortar interface (SEM), and (d) agglomerate of PPFs (SEM).

Fig. 8 shows the micromorphology of fiber–mortar interface. The interface between SF and mortar is obvious, while the wall effect is insignificant, as shown in Fig. 8a. Meanwhile, from the cross-section view shown in Fig. 8b, the SF is closely embedded in the mortar. Therefore, the interfacial effect caused by the incorporation of SF is insignificant. Thus, the interfacial zone around the steel fibers does not act as a preferential path for chloride transport. Therefore, the addition of steel fibers in mortars has insignificant effect on the chloride penetration. As shown in Fig. 8c, the PPF is tightly combined with the mortar, and there is no obvious interface. Nevertheless, the PPFs are dispersed unevenly, and they gather in groups, as shown in Fig. 8d. In addition, the PPF is hydrophobic, and bubbles are inevitably formed during the mixing process.^37^ Consequently, the total porosity of mortar increases gradually with the PPF content increasing from 0 to 0.5%. Besides, the proportion of large pores (200–1000 nm) has an obvious increase when the PPF content comes up to 0.5%.

## 4. Conclusions

The effect of polypropylene fiber and steel fiber content on chloride transport in unsaturated and saturated mortars was investigated in detail. Moreover, the pore structure of fiber reinforced mortars and the micromorphology of fiber–mortar interface were detected.

(1) When the SF content increases from 0 to 1.5%, the chloride diffusion coefficient of mortar has insignificant variation under both of the unsaturated and saturated conditions. This is because the addition of 0–1.5% SF has unremarkable effect on the pore structure of mortars, including pore size distribution and total porosity. Besides, the interfacial effect caused by the incorporation of SF is insignificant.

(2) Compared with the mortars without fibers, the addition of 0.1–0.5% PPF has no obvious effect on the chloride diffusion coefficient, no matter under unsaturated and saturated conditions. However, when the PPF content increases from 0% to 0.5%, the pore size of mortar has a slight decrease while the total porosity increases from 12.55% to 17.67%, mainly caused by the agglomerate of PPFs. With the addition of 0–0.5% PPF, the influence of pore size refinement and porosity improvement on chloride transport counteracts each other.

(3) Overall, with the addition of 0–1.5% SF or 0–0.5% PPF, chloride transport in the mortars has no insignificant variation. However, the chloride diffusion coefficient in unsaturated mortar is an order of magnitude higher than the saturated mortar. The saturation degree is a critical factor influencing the chloride transport.

## Data availability

All data generated or used during the study appear in the submitted article.

## Conflicts of interest

The authors declare that they have no known competing financial interests or personal relationships that could have appeared to influence the work reported in this paper.

## Supplementary Material
